# Dr. Peirce on Dental Education and the St. Louis Meeting

**Published:** 1904-09

**Authors:** C. N. Peirce

**Affiliations:** Philadelphia


### Domestic Correspondence.

### DR. PEIRCE ON DENTAL EDUCATION AND THE ST. LOUIS MEETING.

To the Editor:

Sir,—I have read your several editorials in the August number of the International Dental Journal, and as usual have read them with interest and pleasure, but what came close to my idea were the practical remarks you made in the American Medical Association, Section on Stomatology, when you state that "the higher in a general way you can educate a man the better, but you must have that education properly arranged in order to be of value. The idea to me is pre-eminently absurd to suppose that because a man has an A.B. degree he is better qualified thereby to become a dentist. It is a fallacy. The high-school degree is equally fallacious. What we need in dentistry is an education that will fit a man to become a dentist. To accomplish this he should come up from the mechanical school, through the higher schools, and then reach a point where he can be of value to himself and the community at large. . . . I do not believe that we can make dentists out of all men, whether they have the A.B. degree or whether they have the high-school degree; that is not the point. We must go deeper if we expect to have symmetry in our educational effort. My own idea has been that the poor dentists we have turned out have, as a rule, been men of higher education—not the fault of the higher education, but the fault of a lack of proper symmetry in that education."

I should say a lack of mechanical ability and cultivation of the same. You are not alone in your knowledge of men with the dental degree, who are unfit to practise dentistry. They may pass a satisfactory examination and comply with the requirements for a degree, but for a thorough knowledge of the manipulative skill essential to the successful practice of their profession they are deficient, and sadly so; hence we have in the profession men who would have been brilliant in other industries, but in dentistry they are a failure. It was the recognition of this fact that induced me at Washington to throw what little influence I possessed in favor of three years of eight months. I felt that this period was long enough to test ability, and, if qualified, to make successful practitioners. To this time and qualifications attained, the graduate adds his experience, which makes his reputation and his living.

I must unite with you as to the action of the St. Louis meeting. It certainly had no authority to do more than consider and express its views in certain resolutions; beyond this their action cannot be binding. To say that all rules and parts of rules in conflict with these resolutions be and are hereby repealed, is simply wind. While it may encourage schools to do what they desire, it cannot make legal their action.

C. N. PEIRCE.

PHILADELPHIA, August 4, 1904.

